# HLA-DRB1*15:01 is associated with a reduced likelihood of longevity in northern European men

**DOI:** 10.1186/s13073-025-01554-1

**Published:** 2025-10-20

**Authors:** Nicolás Mendoza-Mejía, Daniel Kolbe, Onur Özer, Janina Dose, Guillermo G. Torres, Andre Franke, Marianne Nygaard, Almut Nebel

**Affiliations:** 1 Institute of Clinical Molecular Biology, Kiel University, University Hospital Schleswig-Holstein, Kiel, Germany; 2 Epidemiology, Biostatistics and Biodemography, Department of Public Health, University of Southern Denmark, Odense, Denmark

**Keywords:** Human leukocyte antigen, Longevity, Genetic association study, Ageing, Immunogenetics

## Abstract

**Background:**

Prior research on the genetics of human longevity has identified only a few robust associations. While these studies highlight the importance of metabolic processes for longevity, the contribution of immune genes, specifically those in the highly polymorphic human leukocyte antigen (HLA) region, remains understudied. Here, we addressed this gap by analysing the influence of HLA variation on longevity in Europeans.

**Methods:**

We conducted an initial case-control study, comparing imputed HLA alleles from a German longevity cohort with younger controls. Associations were evaluated with logistic regression, adjusting for multiple testing and population structure. Subsequently, significant associations (adjusted *P* ≤ 0.05) were tested for replication in two additional populations of similar ancestry: a Danish longevity cohort and the UK Biobank. Furthermore, epitope binding and immunogenicity predictions were performed to detect potential mechanisms linking HLA alleles to longevity.

**Results:**

Our analysis revealed a novel male-specific association of HLA-DRB1*15:01:01 with longevity (adjusted *P* = 2.80 × 10^–2^, odds ratio = 0.64, 95% CI: 0.48–0.82). In Germans, HLA-DRB1*15:01:01 was less frequent among male cases (10%) than controls (15%), whilst female cases exhibited no substantial decrease (14%), suggesting that men carrying this allele have a lower chance of becoming long-lived. This finding was replicated in the UK Biobank and found to be consistent in the Danish cohort. Computational predictions further revealed that HLA-DRB1*15:01 is more likely to trigger an immune response against an apolipoprotein B-100 (APOB-100) epitope than other *HLA-DRB1* alleles. Furthermore, the overall predicted APOB-100 immunogenicity of all *HLA-DRB1* alleles was significantly associated with longevity (estimate −0.11, SE = 0.03, *P* = 0.005).

**Conclusions:**

The novel male-specific association between HLA-DRB1*15:01 and longevity has been observed in three independent cohorts. The anti-longevity effect of this association is perhaps a consequence of an increase in Alzheimer’s disease (AD)-related mortality in men carrying this allele. This hypothesis is based on prior research that has identified a male-specific association between HLA-DRB1*15:01:01 and AD. Additionally, it is likely that this link is mediated by increased immune reactivity against APOB-100, which is promoted by HLA-DRB1*15:01:01.

**Supplementary Information:**

The online version contains supplementary material available at 10.1186/s13073-025-01554-1.

## Background

Human longevity, defined as living beyond the 95th percentile of one’s birth cohort, is influenced by both environmental and genetic factors [1]. Prior genetic studies on long-lived individuals (LLI) have identified numerous associated genetic variants. However, only a limited number have been robustly replicated across various populations, most notably those in *APOE* and *FOXO3* [2–4]. These two genes encode proteins involved in lipid transport and insulin-IGF1 signalling, respectively, underscoring the well-established link between metabolism and longevity. Interestingly, even though immunity is well recognised as a key physiological process in longevity [5, 6], the number of immune-related variants associated with longevity remains scarce.

Immune function has been shown to decline with age, increasing the risk of infections, autoimmune disorders, and cancer [7, 8], which further exacerbate the immune dysfunction [9]. One of the most important immune gene clusters is the human leukocyte antigen (HLA) region because its genes encode molecules responsible for pathogen detection and immune activation. Hence, HLA polymorphisms affect susceptibility to and resistance against more diseases than those from any other region in the human genome [10]. Despite its crucial role, this region has often been superficially analysed due to its extreme polymorphism, high structural variability and the presence of numerous paralogous genes [10]. This complexity renders typing of HLA genes difficult. Consequently, its alleles differ from alleles in most other genes, as they are composed of multiple polymorphisms grouped into known sequences, which derives in a standardised nomenclature (e.g. HLA-A*01:01). This structured classification allows researchers to characterise new samples more effectively, overcoming the challenges of genotyping individual single nucleotide polymorphisms (SNPs) in isolation — the root of HLA’s under-representation in genome-wide association studies (GWAS) [10]. For human longevity, only three prior GWAS identified HLA associations based on SNPs, all of which were located in or near the *HLA-DR/DQ* subregion [11–13].

Early studies relied on serological typing, an approach for genotyping HLA that directly classifies its synthesised protein complexes. Using this technique, two reports in the 1990 s described significant associations between *HLA-DR* serotypes and longevity [14, 15]. However, they have not been replicated in other populations. The lack of replication is largely attributable to the limitations of serotypes, since they represent broad groups of HLA alleles spanning multiple genes, limiting their resolution and interpretability. Consequently, DNA-based HLA typing has since replaced serological methods, offering more precise and reliable allele-level classification. Despite these advances, no study to date has analysed high-resolution HLA alleles (i.e., 2-fields) in the context of longevity. To address this gap, we performed an HLA-longevity study in a German cohort, using allele imputation to generate high-resolution genotypes. We identified a male-specific association of HLA-DRB1*15:01 with reduced longevity, which was subsequently confirmed in the UK Biobank. Additionally, we explored potential mechanisms suggesting that this negative effect may be linked to neurodegeneration.

## Methods

### Study populations

#### German longevity cohort

The primary study population consisted of 1,463 unrelated German long-lived individuals (LLI) and 6,464 geographically matched younger controls. The LLI cohort had a mean age at recruitment of 99.0 years (range: 94–110 years, > 95th age-at-death percentile of birth cohort), including 695 centenarians, with an overall female-to-male ratio of 2.75:1. Additional recruitment criteria for LLI included the absence of overt signs of major diseases and overall good health and mental status [16]. The younger control group had a mean age of 57.2 years (range: 18–83 years) with a female-to-male ratio of 1.05:1. To ensure a population-representative sample, the inclusion of controls was not based on the presence or absence of any specific disease. The project was approved by the Ethics Committee of the Medical Faculty of Kiel, and written consent is available from all participants.

#### Danish longevity cohort

An independent Danish longevity cohort was used to replicate the significant findings from the German cohort. Cases included individuals older than 95 years at death/censoring (*N* = 800, 76.4% female, mean age at death/censoring = 100.7 years) from the 1905, 1910, 1911–1912, and 1915 birth cohort studies [17, 18], the Study of Danish Old Sibs, and the Longitudinal Study of Ageing Danish Twins (LSADT) [19]. Controls included individuals younger than 90 years at death/censoring (*N* = 1148, 47.3% female, mean age at death/censoring = 78.8 years) from the study of Middle-Aged Danish Twins (MADT) [19]. From LSADT and MADT, one individual from each monozygotic twin pair and both twins from dizygotic twin pairs were included.

Individuals were followed until death or until December 1^st^, 2023, whichever came first. At the time of censoring, approximately 73% of controls were still alive (mean age 78.8 years, range: 70.9–89.8 years), while the remaining 27% had died before the age of 90 (mean age at death: 78.9 years, range: 60.1–89.9 years). Controls who were older than 90 years at last follow-up were excluded (*N* = 54, still alive 76%), as they could potentially reach the longevity threshold of 95 years, making their status uncertain.

#### The UK Biobank

Imputed HLA alleles from the UK Biobank were used to replicate the significant HLA associations observed in the German cohort. Alleles with *r*^2^ ≤ 0.7 were excluded as recommended by the UK Biobank guidelines. Allele homozygosity was determined based on its predicted abundance.

Given that offspring of long-lived parents inherit a combination of genetic variants that enabled their parents to achieve longevity, they tend to live longer than offspring of shorter-lived parents and show a lower incidence of age-related diseases [20–22]. Therefore, parental longevity has been employed as a valuable proxy for studying healthy ageing in several prior studies [11, 12, 23]. We analysed the age distribution and defined 95 years of age (99th percentile) as the threshold for parental longevity (Additional file 1: Fig. S1). Hence, individuals with parents older than 95 years (long-lived parents) were defined as cases. In contrast, individuals whose parents had an unknown age at death or died before 40 years were excluded to reduce the influence of non-natural causes of death like accidents and war, as in previous longevity studies [23]. Controls were defined as individuals with parents aged 90 years or younger.

### Immunochip dataset

The individuals of the German longevity cohort were genotyped using the Immunochip (Illumina, Inc., CA). These data were previously published by Flachsbart et al*.* [3], with additional cohort descriptions. Genotype data included 4,504 SNPs within the HLA region, with an average GenTrain score of 0.83. We performed quality control on the data using the plinkQC package [24]. In short, we removed outliers based on the missing genotype rate (> 0.03), heterozygosity rates (*Z* > 3 or *Z* < −5) and relatedness (identity-by-descent > 0.18). We also excluded individuals with divergent ancestry, which was determined by principal component analysis (PCA) anchored with HapMap 3 as the reference population. Finally, we conducted per-marker quality control, removing single-nucleotide variants with excessive missingness rates (> 0.01), significant deviations from Hardy–Weinberg equilibrium (*P* < 1×10^–05^), and low minor allele counts (< 20).

### Allele imputation

Using the quality-controlled genotype dataset described in the previous section, HLA alleles were imputed with HLA-TAPAS [25], an updated and enhanced version of SNP2HLA [26], known to provide improved accuracy and resolution. We utilised the 1000 Genomes reference panel provided within HLA-TAPAS. From the HLA-TAPAS imputation output, we kept only HLA alleles with an *r*^2^ > 0.8 and an allele frequency > 1% in our samples, yielding 146 HLA class I and II alleles. We then formatted the alleles with vcf2allele [27] for downstream analyses. The vcf2allele algorithm uses both genotype and dosage information to assign alleles. Therefore, the *r*^2^ threshold was applied beforehand to ensure reliable genotype calls. For readability, we will omit the “HLA-” prefix when referring to specific HLA alleles.

### Association analysis

Association analyses were conducted using the pyHLA software package [28]. For initial allele-phenotype association testing, we employed logistic regression under an additive genetic model. To ensure sufficient statistical power to detect associations with an odds ratio (OR) ≥ 1.5, given our sample size, only alleles with a frequency greater than 5% were included in the analysis to maintain statistical robustness. Additionally, to control for potential confounding due to population structure, the top three principal components (PCs) obtained during quality control were included as covariates in the regression models. The number of PCs was determined via the scree plot approach. Significance levels were corrected using the Bonferroni method to account for multiple testing and additional sex stratification.

To further dissect the complex genetic architecture of the HLA region and explore potential interactions between HLA alleles as well as between HLA and *APOE*, we utilised an established interaction test specific for HLA alleles [29, 30]. This pairwise association approach, implemented in pyHLA, analyses allele pairs using eight distinct statistical tests, each based on 2 × 2 contingency tables. Significance for each test is determined using Fisher’s exact test to evaluate independence, synergistic/antagonistic interactions, differential associations, combined action, and linkage disequilibrium. Further details of this pairwise testing are provided in Supplementary Methods S1 (Additional file 1). Additionally, to assess epistasis beyond additive allele interactions, we performed a separate analysis using the “--fast-epistasis boost” function in PLINK, considering results with *P* > 0.05 as indicative of no significant epistasis.

Specifically, we performed pairwise testing on significantly associated HLA alleles and examined interactions between DRB1*15:01 and both *APOE ɛ4* and *APOE ɛ2* using UK Biobank data. While we acknowledge the potential for interactions with other longevity-associated variants such as those in *FOXO3* and *CDKN2B* genes [3], we focused on specific *APOE* alleles for practical and biological reasons: *APOE ɛ4* and *APOE ɛ2* have a relatively common allele frequency in European populations (15.5% and 6.6%, respectively [31]), which makes interaction testing feasible within large cohorts like the UK Biobank. In contrast, detecting interactions involving other longevity-associated variants, which may have smaller effect sizes or lower frequencies, might require prohibitively large sample sizes. Furthermore, from a biological perspective, both *APOE* and HLA genes (specifically *DRB1*) are implicated in neurodegeneration [32], making their interaction plausible, especially because the two *APOE* alleles are known to have opposing effects on disease risk. Finally, it is important to note that while the UK Biobank is not specifically a cohort of long-lived individuals, its large sample size and extensive phenotyping data provide robust statistical power for interaction testing.

#### Replication in the Danish longevity cohort

To replicate the significant finding observed in Germans, we analysed an independent Danish longevity cohort. Here, genotypes of rs3135388, a tag SNP for DRB1*15:01, were extracted from available microarray data (from the Illumina Infinium PsychArray or the Illumina Human OmniExpress Array). Subsequently, a sex-stratified association analysis was performed using a logistic regression model with twin pair number as a random effect to account for the relatedness between twins from the same twin pair. The use of logistic regression ensured that the analytical method was consistent across all cohorts, ensuring the results were directly comparable.

#### Replication in the UK Biobank

The UK Biobank was used to replicate the significant HLA association observed in the German cohort. This resource had imputed HLA alleles readily available. A sex-stratified logistic regression model was performed using individuals with a long-lived father (*n* = 4747) or mother (*n* = 11,941) as cases and individuals without a long-lived father (*n* = 318,421) or mother (*n* = 242,100) as controls. To correct for population stratification, we included only individuals who self-reported as white or British and adjusted for the first three principal components, as done for the German cohort. The association analysis was otherwise identical to that of the main dataset and is detailed in the “Association analysis” section.

### Validation of DRB1*15:01/DRB1*15:01:01 imputation

To validate the DRB1*15:01:01 imputation, we employed two strategies. First, to assess the imputed allele frequency in controls, we compared the frequency of the 2-field DRB1*15:01 allele in controls with the reported frequency of the rs3135388-A allele*.* rs3135388 is a tag-SNP known to be in strong linkage disequilibrium with DRB1*15:01 in Europeans (*r*^2^ = 0.97) [33, 34]. The allele frequency of rs3135388-A for CEU (Northern Europeans from Utah) was obtained from Ensembl [31].

Second, to directly evaluate imputation performance in cases, we performed targeted HLA sequencing (HLA-seq) on 100 randomly selected LLI imputed to carry DRB1*15:01:01 and 100 LLI imputed not to carry this allele. HLA-seq was applied to obtain highly accurate HLA genotypes. Genomic DNA was extracted using the QIAamp DNA Blood Mini Kit (Qiagen) according to the manufacturer’s instructions. High-resolution HLA typing was performed using a targeted NGS method as described by Wittig et al*.* [35]. Briefly, genomic DNA was randomly fragmented, followed by DNA library preparation using the NEBNext® Ultra™ DNA Library Prep Kit for Illumina® (New England Biolabs, Ipswich, MA, USA). The HLA target was enriched with a biotinylated RNA bait library using a commercially available enrichment kit (myBaits Custom 1–20 K; BioCat GmbH, Heidelberg, Germany). The enriched DNAs were sequenced on the Illumina NovaSeq 6000 platform (2 × 150 bp read length) at the Competence Centre for Genome Analysis Kiel (CCGA Kiel). HLA alleles were genotyped from the sequencing reads using five software packages from the Institute of Clinical Molecular Biology (IKMB) pipeline [36]. A custom voting algorithm [27] was applied to combine these results into a consensus allele, enhancing genotyping accuracy. Imputation performance was assessed by comparing HLA-seq genotypes with imputed DRB1*15:01:01 alleles, measuring precision and recall.

### Computation of effect size variation

Effect sizes were compared across cohorts to assess whether they differed significantly. The *Q*-statistic was calculated and a *p*-value < 0.05 was considered evidence against the null hypothesis of a shared true effect size. The *I*^2^ statistic was computed to estimate the proportion of observed variance attributable to heterogeneity in the true effects. These statistics were calculated using the PythonMeta package [37]. Initially, effect sizes for German and Danish males were compared, as both cohorts directly measure longevity and represent genetically similar populations. This comparison aimed to assess variation in effect size under direct longevity assessment. Subsequently, effect sizes from all three cohorts, including the UK Biobank, were analysed to evaluate differences when using paternal age as a proxy for longevity.

### Assessment of potential confounders

Given the known association of DRB1*15:01 with multiple sclerosis (MS) risk and the fact that the SNP rs9267649 has been reported to mitigate this risk by influencing *DRB1* gene expression through methylation [38], we assessed if rs9267649 was a potential confounder in our longevity analysis. Correlation was calculated using PLINK and including only rs9267649 with logistic regression with an additive allele model. Linkage disequilibrium was calculated in the 1000 Genomes CEU population using the online tool LDpop [39].

### Assessment of peptide binding affinity and presentation

Binding affinity data for DRB1*15:01 were retrieved from the Immune Epitope Database (IEDB) [40]. We collected all available peptide binding assays for both pathogen-derived and self-antigens. The data were grouped by genus and protein name for pathogens and self-antigens, respectively. To minimise bias from underrepresented epitopes, only groups with more than 10 reported assays were included. Assay results were categorised into five binding affinity levels: high, intermediate, binding, low and no binding.

While the IEDB is a widely used resource, its self-antigen binding reports are inherently biased towards antigens that have established associations with autoimmune diseases. Additionally, for an immune reaction to occur, not only must the peptide bind to HLA, but it must also be successfully presented to T-cell receptors (TCR). Therefore, we performed a more systematic assessment based on computational predictions to minimise this bias and obtain a more reliable metric of immune reactivity against self-epitopes. We generated binding affinity predictions for all epitopes known to bind to *DRB1* molecules, followed by an immunogenicity prediction to evaluate the likelihood of triggering an immune response. We focused on *DRB1* alleles with frequencies above 5% in our German cohort to ensure statistical power. For all *DRB1* alleles, we collected all binding assays on linear epitopes (continuous sequence of amino acids) reported in the IEDB. Only epitopes with a length of 13–25 amino acids were included, as peptides that bind to class II molecules are predominantly within this range [41]. Binding predictions were performed using NetMHCIIpan [42], a validated tool for MHC-II binding prediction. Epitopes for which DRB1*15:01 exhibited the highest binding affinity were selected. The immunogenicity of these epitopes in DRB1*15:01 carriers was then predicted using TLimmuno2 [43]. Given that this tool ranks the epitope-HLA binding against a dataset of 13- to 21-mer peptides, epitopes longer than 21 amino acids were excluded from this last step of the analysis. Immunogenicity ranks below 0.1 and predicted immunogenicity scores above 0.90 were considered likely to trigger an immune response. This set of proteins was used in the next analysis.

### Association analysis between immunogenicity and longevity

To determine whether the link between *DRB1* alleles and longevity is mediated through increased immune reactivity against self-epitopes, we used logistic regression to assess whether longevity status was associated with predicted immunogenicity scores in the German cohort. Epitopes originating from the four most immunogenic proteins in DRB1*15:01 carriers (determined in the previous section) were collected from IEDB. TLimmuno2 [43] was used to predict the immunogenicity scores, this time, of the most common *DRB1* alleles (> 5% frequency) against this subset of epitopes. The resulting immunogenicity scores were mapped to each study participant based on their specific *DRB1* allele. For heterozygous individuals, the average of the two alleles was taken. This strategy was used because DRB1 alleles are codominant. Therefore, each allele in a heterozygous individual is expressed at half the level compared to a homozygote. Consequently, if we assume that the probability of recognising a given epitope scales with the expression level (a strategy also used to train TLimmuno2), the immunogenicity score of an epitope in a heterozygous individual could be approximated by the average of the two allele-specific scores.

Additionally, given that there were multiple epitopes per protein, we selected the epitope with the maximum predicted immunogenicity score to represent the protein. Our assumption was that the epitope with the highest likelihood for successful antigen presentation would initiate the immune response before other epitopes derived from the same protein. Finally, we performed a logistic regression analysis. Longevity status (long-lived vs. non-long-lived) served as the dependent variable, and the immunogenicity scores for each protein were used as independent variables in the model. The model was fitted using robust maximum likelihood (MLR estimator from the R library lavaan [44]), which provides heteroscedasticity-consistent standard errors and non-normality of residuals. We compared standard maximum likelihood estimation with heteroscedasticity-robust (Huber-White) standard errors to assert adjustments for heteroscedasticity if present. Independent variables with a variance inflation factor (> 12) were excluded. Correction for population stratification was done by including the first three principal components calculated during the quality control stage, as described above in the “Immunochip dataset” section. Sex was also included as a covariate to account for sex-specific effects. Finally, the method “check_model” from the R library “performance” [45] was used for the visual assessment of the model assumptions.

## Results

After quality control (Additional file 1: Figs. S2, S3) and imputation, alleles from three HLA class I genes (*A*, *B* and *C*) and five class II genes (*DPA1*, *DPB1*, *DQA1*, *DQB1* and *DRB1*) were determined. The dataset included 7785 individuals, comprising 1453 LLI (mean age 99.0 years) and 6332 younger controls (mean age 57.2 years). The number of samples genotyped for each gene is shown in Additional file 2 (Table S1). We were able to impute HLA alleles at the 3-field resolution (Additional file 2: Table S2). On average, each sample had 15.68 alleles at this level, out of 16 possible (8 loci, two alleles per locus) (Table 1).

**Table 1:** Number of alleles by resolution. Number of HLA alleles classified at different resolution levels for the 7785 study participants. The 1-field resolution represents broader allele groups, while the 2-field resolution identifies specific protein variations. The 3-field resolution provides the highest granularity, distinguishing synonymous mutations within coding regions

Resolution	Number of alleles
1-field	1285
2-field	351
3-field	122,092

### DRB1*15:01:01 was negatively associated with longevity in men

Next, we performed an association test for all HLA alleles at the 3-field resolution. Although no allele was statistically significant after multiple testing for the whole population, DRB1*15:01:01 reached significance when stratifying by sex (Table 2, Additional file 2: Table S5). In the male subgroup (388 LLI vs. 3,150 controls), the presence of DRB1*15:01:01 was associated with a lower likelihood of becoming long-lived (OR = 0.63, adj. *P* = 2.80 × 10^–2^, 95% CI: 0.48–0.82). No significant difference was observed between individuals carrying DRB1*15:01:01 in the homozygous or heterozygous state (*P* = 7.21 × 10^–2^). Given the pleiotropic effects of multiple HLA alleles on the same phenotype, we performed a pairwise association test to investigate potential interactions between allele pairs. The results revealed that the association between DRB1*15:01:01 and longevity was linked to DRB1*15:01:01 ~ DQB1*06:02:01 (OR = 0.64) — a well-known haplotype in Europeans that encodes the DR15 serotype [46, 47].

**Table 2:** Results of sex-stratified HLA association tests for DRB1*15:01:01. Separate analyses were conducted for males, females, and the combined study population. A significant association was only observed in males (highlighted in bold). Adjusted *p*-values were calculated using Bonferroni correction, accounting for the number of genotyped alleles (> 5% frequency) and the number of sex-stratified tests

	DRB1*15:01:01		
Population	Males	Females	Whole study population
Allele frequency in LLI	0.10 (*n* = 62)	0.14 (*n* = 251)	0.13 (*n* = 313)
Allele frequency in controls	0.15 (*n* = 778)	0.15 (*n* = 786)	0.15 (*n* = 1564)
*p*-value	7.03 × 10^–04^	0.74	0.04
Adjusted *p*-value	**2.80 × 10**^**–2**^	1	0.82
Odds ratio	**0.63**	0.97	0.87
95% confidence interval	0.48–0.82	0.84–1.13	0.77–0.99

In a further explorative analysis, we assessed whether the link of DRB1*15:01:01 with longevity is stronger in centenarians by performing association tests restricted to male (*n* = 137) and female (*n* = 555) centenarians, respectively. The ORs between centenarians and LLI (data not shown) differed only slightly and were not significant after correction for multiple testing.

### Imputation of DRB1*15:01/DRB1*15:01:01 was reliable

The reliability of the DRB1*15:01:01 imputation was assessed with two validation strategies. First, we compared the imputed allele frequency in controls with the reported population allele frequencies by using a tag-SNP. Specifically, we used the rs3135388-A allele, a well-known proxy for the 2-field resolution allele DRB1*15:01 (with a strong linkage disequilibrium in Europeans; *r*^2^ = 0.97) [33, 34]. The reported frequency of the rs3135388-A allele in northern Europeans (16%, [31]) closely matched the imputed frequency of DRB1*15:01 in our controls (15.03%). Second, to directly assess imputation accuracy in cases, we performed high-resolution HLA sequencing (HLA-seq) on 100 German LLI imputed to carry DRB1*15:01:01 and 100 LLI imputed not to carry this allele. A comparison of the imputed genotypes with the highly accurate HLA-seq data (Additional file 2: Table S3) revealed that the imputation of DRB1*15:01:01 was highly reliable (Additional file 2: Table S4), with 93.33% precision, 100% specificity and 97.68% recall.

### DRB1*15:01 association was supported by findings in two additional cohorts

**Fig. 1:**
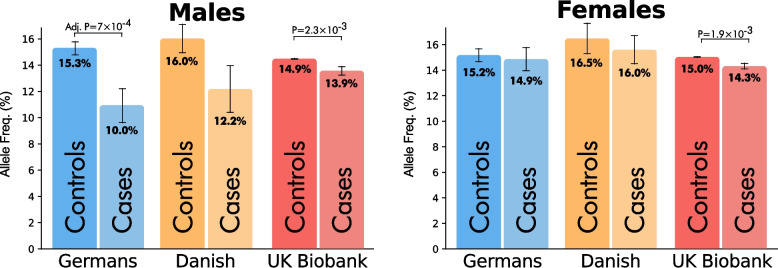
Sex-stratified DRB1*15:01 allele frequencies across cohorts. DRB1*15:01 allele frequencies are presented for German, Danish and UK Biobank cohorts. German and UK Biobank frequencies are based on imputed alleles, while Danish frequencies are based on the A-allele frequency of the tag-SNP rs3135388. Germans: cases ≥ 95 years, controls ≤ 83 years; Danish: cases ≥ 95 years, controls < 90 years; UK Biobank: cases are children of parents with age at death ≥ 95 years (father, left; mother, right), controls are children of parents with age at death between 40 and 90 years (father, left; mother, right). The adjusted *p*-value was calculated using Bonferroni correction, accounting for the number of genotyped alleles (> 5% frequency) and the number of sex-stratified tests

The significant association identified in the German study population was followed up in a Danish longevity cohort and the UK Biobank. First, we examined a Danish longevity cohort using the tag-SNP rs3135388-A as a proxy for DRB1*15:01. In Danish men, we observed a trend (OR = 0.74, *P* = 0.07, 95% CI: 0.51–1.02, based on 189 LLI and 605 controls), mirroring the direction of the association in German males (Fig. 1, Additional file 2: Table S6). Second, we analysed imputed HLA alleles (at 2-field) from the UK Biobank, using individuals with a long-lived father (age at death ≥ 95 years, *n* = 4747) or mother (*n* = 11,941) as cases and individuals without a long-lived father (*n* = 318,421) or mother (*n* = 242,100) as controls. DRB1*15:01 showed a significant association with reduced odds of both paternal (adj. *P* = 2.30 × 10^–2^, OR = 0.92, 95% CI: 0.87–0.98) and maternal longevity (adj. *P* = 1.04 × 10^–2^, OR = 0.94, 95% CI: 0.91–0.98). The lower effect sizes of DRB1*15:01 in the UK Biobank, compared with the other cohorts, were presumably a consequence of using paternal or maternal age at death as a proxy for longevity. Collectively, the consistency of the DRB1*15:01 association across Germans, Danish and UK Biobank cohorts further supports the potential role of DRB1*15:01 in longevity, suggesting that this association holds true in other northern European populations.

### The inclusion of UK Biobank introduces heterogeneity in the effect sizes

The heterogeneity of effect sizes of DRB1*15:01 on longevity across the different cohorts was calculated to evaluate their consistency. Initially, we assessed heterogeneity between the German and Danish cohorts, since both used direct measures of longevity and share similar genetic backgrounds. Under these conditions, no heterogeneity was observed (OR = 0.66; Tau^2^ = 0; *Q* = 0.72, *P* = 0.397; *I*^2^ = 0%), indicating that the effect of DRB1*15:01 on longevity remains strong in populations of similar ancestry.

The subsequent inclusion of the UK Biobank, which uses parental age at death as a proxy for longevity, introduced substantial heterogeneity (OR = 0.77, prediction interval: 0.55–0.98; Tau^2^ = 0.046; *Q* = 9.27, *P *= 0.01; *I*^2^ = 78.42%). Hence, the increased heterogeneity likely reflects differences in phenotype (longevity vs. paternal age at death) rather than conflicting associations. Overall, all studies showed a consistent direction of effect, with DRB1*15:01 associated with reduced likelihood of achieving longevity.

### The association was independent of *APOE* variants

Additionally, since both DRB1*15:01 and *APOE ε4*, a major anti-longevity allele, have been associated with Alzheimer’s disease (AD) pathology [32], we investigated potential interactions between DRB1*15:01 and *APOE* status in the UK Biobank. Pairwise analysis revealed that the associations of *APOE ε4* (*P* = 4.17 × 10^–13^, OR = 0.79, 95% CI: 0.75–0.84) and DRB1*15:01 (proxy SNP rs3135388-A *P* = 1.97 × 10^–4^, OR = 0.89, 95% CI: 0.83–0.94) with longevity were independent of each other and exhibited an additive effect in carriers of both alleles (*P* = 1.54 × 10^–10^, combined OR = 0.70, 95% CI: 0.62–0.78). In contrast, the combination of the pro-longevity allele *APOE ε2* and DRB1*15:01 showed a neutral combined effect (*P* = 0.97, combined OR = 0.99, 95% CI: 0.87–1.14). However, no epistasis was observed.

### DRB1*15:01-MS association did not confound our longevity findings

To identify potential factors that might have influenced the observed association between DRB1*15:01 and longevity, we considered other phenotypes strongly associated with this HLA allele. In this regard, literature consistently reports a robust link between DRB1*15:01 and multiple sclerosis (MS) risk [34, 38, 47, 48], positioning MS as a primary candidate for a potential confounder. This was further supported by a phenome-wide association study (PheWAS) [49], which identified associations with several diseases, including systemic lupus erythematosus (*P* = 0.002). These associations may explain the increased mortality observed in carriers vs. non-carriers. However, MS risk still showed by far the strongest association (*P* = 8.03 × 10^–24^; Additional file 1, Fig. S4).

Given the strong prior association between DRB1*15:01 and MS, we investigated whether this relationship could confound our longevity findings. Specifically, we examined the role of the SNP rs9267649, which has been reported to influence *DRB1* gene expression and mediate the risk of MS associated with DRB1*15:01 [38]. Our analyses revealed no significant association between rs9267649 and longevity (*P* = 0.75, OR = 0.98, 95% CI: 0.86–1.11), and no evidence of high linkage disequilibrium between rs9267649 and DRB1*15:01 (*r*^2^ = 0.43, *D*′ = 0.84). Even after excluding carriers of the risk allele for MS (rs9267649-G), the frequency of DRB1*15:01 remained lower in LLI compared to controls (allele frequency = 42.8% in LLI vs 67.0% in controls).

### DRB1*15:01 was unique due to its high immunogenicity to myelin and APOB-100

Given the strong association between DRB1*15:01:01 and longevity, we aimed to identify potential epitopes from bacteria, viruses and self-antigens that could trigger immune responses in individuals carrying this allele. For this purpose, we used the Immune Epitope Database (IEDB) [40]. It compiles results from binding assays, a technique that measures the ability of HLA molecules to bind peptide sequences (epitopes), thus giving insights into which antigens are likely to be recognised and presented by the immune system. Since IEDB provides binding assay data for alleles up to 2-field resolution, we used the 2-field version of DRB1*15:01:01 for our analyses.

**Fig. 2:**
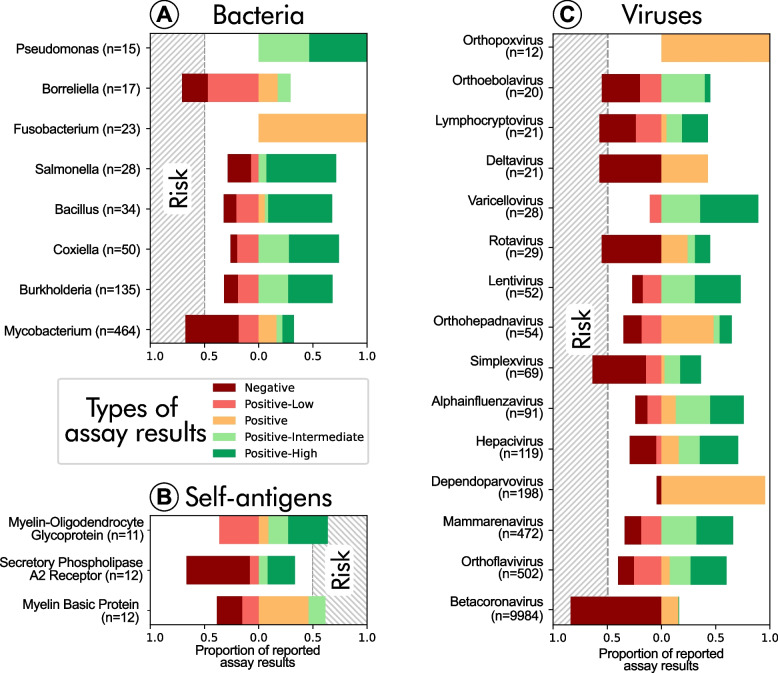
DRB1*15:01 binding affinities across epitope categories. **A** Bacterial epitopes by genus. **B** Self-antigens by protein name. **C** Viral epitopes by genus. Data, sourced from the Immune Epitope Database (IEDB), are visualised as stacked bar charts. Only groups with more than 10 reported assays are shown. Each bar represents 100% of tested epitopes within a category with colour-coding indicating binding strength (negative: red; positive-low: orange; positive: light green; positive-intermediate: green; positive-high: dark green). Grey areas in panels **A**–**C** highlight categories where positive binding reports exceed 50% suggesting a potential disease risk

Overall, DRB1*15:01 was able to recognise a broad range of bacterial and viral genera (Fig. 2A, B). For instance, it exhibited a high binding affinity for the genus *Alphainfluenzavirus* (Fig. 2B), consistent with its reported protection against influenza A [50]. Yet, this allele is not universally protective, as its carriers are susceptible to some infections, such as *Mycobacterium leprae* [51], which correlates with its low binding affinity for *Mycobacterium* (Fig. 2A). Interestingly, while DRB1*15:01 shows low binding affinity for the *Mycobacterium* genus overall, this allele has been reported to confer protection against tuberculosis [52]. Regarding self-antigens, two myelin sheath proteins had the most reported assays (Fig. 2C), both showing high binding affinity, which might potentially contribute to an autoimmune-like response.

The reported assays for self-antigens may be biased by prior associations as epitopes suspected to influence a given condition are more likely to be tested and published. Moreover, the high binding affinity of an HLA molecule to a self-antigen does not necessarily induce an immune response as additional factors, such as T-cell receptor (TCR) interactions, contribute to this process [43]. To address these limitations, we implemented a two-step computational prediction approach to identify self-antigens that could trigger an autoimmune response in DRB1*15:01 carriers and thus potentially lead to an anti-longevity effect. First, we predicted binding affinities for all *DRB1* alleles with a frequency above 5% in our German population (Additional file 2: Table S7). As a result, we obtained a list of 15 epitopes for which DRB1*15:01 exhibited the highest affinity among all tested *DRB1* alleles. Second, we performed immunogenicity predictions on these epitopes. Based on the predictions, myelin had the highest immunogenicity, which is consistent with the strong association between DRB1*15:01 and demyelinating diseases like MS. Three additional epitopes were predicted to likely promote an immune response in DRB1*15:01 carriers (Additional file 2: Table S8). Notably, an epitope from apolipoprotein B-100 (APOB-100) — a key protein in lipid transport and a biomarker for longevity [53] — was part of these four highly immunogenic peptides.

#### Predicted immunogenicity against APOB-100 was associated with longevity

Four self-antigens, identified in the previous section, were predicted to be highly immunogenic exclusively for DRB1*15:01. Therefore, to assess whether specific *DRB1* alleles, like DRB1*15:01, might influence longevity by increasing the likelihood of an immune reaction against this subset of epitopes, we investigated the link between computationally predicted immunogenicity (Additional file 2: Table S9) and longevity status within the German cohort. To account for allele-specific effects, we predicted immunogenicity for all DRB1 alleles against epitopes derived from these four self-antigens. Predictions were mapped to each individual based on their genotyped alleles. Subsequently, logistic regression models were estimated using maximum likelihood with robust standard errors, revealing a significant association between the increase of immunogenicity against APOB-100 and a reduced likelihood of reaching longevity (estimate = −0.11, robust SE = 0.03, *P* = 0.005; Additional file 2: Table S10). Notably, after excluding carriers of DRB1*15:01, the association was no longer significant for APOB-100 (*P* = 0.87), indicating that this allele was the main driver of the association. Furthermore, we verified that the logistic regression model met the required assumptions (Additional file 1: Fig. S5). Overall, our findings suggest that an increased immune reactivity towards APOB-100 contributes to the observed association between DRB1*15:01 and longevity.

## Discussion

In this study, we analysed high-resolution HLA alleles imputed from a German cohort to investigate their potential association with human longevity. We identified a novel sex-specific association with DRB1*15:01:01, which reduces the likelihood of achieving longevity in men. A similar trend was observed in a Danish cohort using rs3135388 as a tag-SNP; however, it was not significant, presumably due to the smaller sample size. This finding was robustly replicated in the UK Biobank at 2-field resolution (DRB1*15:01) using both paternal and maternal age at death (≥ 95 years) as longevity proxies (Fig. 1). In females, a small trend was noticeable in the German and Danish cohorts. While it was consistently weaker than the effect in males, the female trend reached statistical significance in children of long-lived mothers in the UK Biobank, likely due to the larger sample size. Overall, our findings indicate that the association of DRB1*15:01 with longevity is predominantly male-specific, aligning with previously reported sex-dependent effects of the DR15 serotype (males: OR = 0.58, females: OR = 1) [14]. Unlike earlier serotype-based studies, which could not resolve allele-level differences because DR15 is encoded by either DRB1*15:01 ~ DQB1*06:02 or DRB1*15:02 ~ DQB1*06:01, our approach allowed us to fine-map the association to the specific haplotype DRB1*15:01:01 ~ DQB1*06:02:01 at 3-field resolution. Although HLA imputation infers alleles from surrounding genetic markers rather than direct measurement and may be affected by reference population biases, our assessment of the impact of these limitations indicated that this technique is highly reliable for typing DRB1*15:01:01. Its high accuracy stems from a strong linkage disequilibrium with rs3135388.

The primary known association of DRB1*15:01 is with MS (Additional file 1, Fig. S4) [38, 49], where it is implicated in driving myelin-specific autoimmunity and neuroinflammation leading to demyelination [54], due to its high binding affinity to this protein (Fig. 2C). However, several factors suggest that the link with MS does not explain the reduced odds of achieving longevity. First, MS incidence is generally higher in women than in men [48] and, particularly, female DRB1*15:01 carriers have a greater risk for MS [55], whereas our association is specific to males. Second, our observation is independent of rs9267649, which mediates the risk of MS driven by DRB1*15:01 [38]. Third, MS has a relatively early onset and its prevalence decreases with advancing age [48]. Therefore, while DRB1*15:01’s role in MS highlights its potential to promote autoimmune and inflammatory processes in the central nervous system, MS is unlikely to explain the observed reduced male longevity. Instead, DRB1*15:01’s negative influence on longevity might stem from other phenotypes.

Within the HLA-DR locus, the DRB1*15:01 allele shows a robust association with late-onset AD [32, 56, 57]. Unlike other neurodegenerative diseases, including MS, AD has been identified through Mendelian randomisation as one of the causal factors preventing individuals from achieving longevity [58]. This relationship explains why genetic variants that predispose individuals to AD, notably *APOE ε4*, also reduce the likelihood of achieving longevity [9]. Interestingly, the previously observed association of DRB1*15:01 with the risk of AD was also male-specific (*P* = 9.6 × 10^–3^, OR = 1.15, 95% CI: 1.03–1.27) [56]. In addition, carriers of this allele have been shown to exhibit a greater rate of cognitive decline and higher baseline levels of AD-related inflammation [56]. Therefore, men carrying DRB1*15:01 probably have reduced odds of reaching longevity due to the increased risk of developing AD.

Furthermore, our computational predictions revealed that DRB1*15:01 (among all tested *DRB1* alleles) exhibited the highest predicted binding affinity (Additional file 2: Table S7) as well as a high predicted immunogenicity against APOB-100 (Additional file 2: Table S8). Interestingly, increased APOB-100 immunogenicity, based on all *DRB1* alleles, was associated with decreased odds for longevity. These findings imply that DRB1*15:01 is capable of triggering an APOB-targeted immune response, potentially promoting inflammation, an effect that has been previously reported [59]. Notably, neurodegenerative inflammation related to APOB-100 has also been observed in mouse models of AD [60]. Given that elevated APOB-100 levels are also associated with both AD [61] and increased mortality [62, 63] in humans, we propose that DRB1*15:01 contributes to the development of AD and a lower likelihood for longevity via immunogenicity against APOB-100. What is more, this may explain the worse disease progression and higher levels of inflammation markers in AD patients carrying DRB1*15:01 [56]. However, we currently lack direct evidence showing that APOB-100 is the specific epitope presented via HLA in this context. In addition, we acknowledge that there might be other, yet unknown, pathophysiological mechanisms contributing to the negative effect of DRB1*15:01.

From an evolutionary perspective, the persistence of DRB1*15:01 in the European gene pool, despite its association with certain diseases, provides evidence for potential selective advantages, particularly in younger individuals. These advantages may include protection against infections (Fig. 2A and B). The allele is hypothesised to have been under selection in Caucasus hunter-gatherers [64], one of the main ancestral populations of steppe herders who later introduced it into the European gene pool [65]. Interestingly, *APOE ε4* also has a hunter-gatherer origin [66]. In the steppe population, DRB1*15:01 may have conferred protection against zoonotic pathogens associated with pastoralist lifestyles and dairy consumption [67]. Indeed, DRB1*15:01 is linked to protection against tuberculosis [52], bacterial gastric diseases [68] as well as influenza A [50] and asymptomatic SARS-CoV-2 infection [69, 70]. Thus, our findings appear to exemplify antagonistic pleiotropy, where an allele beneficial in early life, possibly by enhancing immune responses to pathogens, becomes detrimental later by increasing the risk of chronic inflammation and age-related decline.

## Conclusions

Our multi-cohort study across German, Danish and UK Biobank populations has uncovered a significant negative association between the HLA allele DRB1*15:01 and longevity in men. We propose that this effect is driven by immune reactivity against APOB-100, which has been shown to accelerate AD progression. Given the known male-specific link between DRB1*15:01 and AD, it is likely that the reduced longevity odds observed in male allele carriers result from increased AD-related mortality. Overall, these findings, together with other longevity markers, may form the basis for new predictive models of longevity and could inform personalised medicine approaches aimed at mitigating age-related mortality risks.

## Supplementary Information

Additional file 1: Contains the supplementary figures and methods. These include figures of distribution of parental age at death in the UK Biobank (Fig. S1); results of the quality control on individuals and markers (for the German dataset; Figs. S1, S2); other phenotypes associated with DRB1*15:01 identified by PheWAS (Fig. S3) and diagnostic plots for the immunogenicity logistic regression model (Fig. S4). The file also includes a description of the interaction tests (Methods S1) and two tables supplementing related to it (Tables S11, S12).

Additional file 2: Contains the supplementary tables. These tables include the number of samples genotyped for each HLA gene (Table S1); imputed HLA allele frequencies from German LLI (Table S2); HLA-seq derived allele frequencies from 200 German LLI (Table S3); a confusion matrix comparing imputed *DRB1* alleles with those obtained from HLA-seq (Table S4); results of the sex-stratified HLA association tests (Table S5); statistics of the DRB1*15:01 association with longevity in the three cohorts (Table S6); predicted binding affinities for all tested *DRB1* alleles (Table S7); highly immunogenic epitopes in DRB1*15:01 carriers (Table S8); predicted immunogenicity scores for all *DRB1* alleles against the four most immunogenic proteins (Table S9); and the results of the logistic regression analysis of immunogenicity scores and longevity (Table S10).

## Data Availability

The Immunochip genotype data (German cohort) used in this study were previously published by Flachsbart et al. [3] and are available upon request from the PopGen Biobank (Schleswig–Holstein, Germany; https://www.uksh.de/p2n/Information+for+Researchers.html). Imputed HLA allele frequencies from German LLI and HLA-seq derived allele frequencies from 200 German LLI individuals are available in the Additional file 2 (Tables S2 and S3, respectively). The data from the Danish Twin Registry (https://www.sdu.dk/en/forskning/dtr) can be accessed upon request; please see https://www.sdu.dk/en/forskning/dtr/researcher. For access to data from the Danish birth cohorts of nonagenarians and centenarians (the 1905, 1910, 1911–1912, and 1915 birth cohort studies, https://www.sdu.dk/en/forskning/darc), please contact Dr. Marianne Nygaard, mnygaard@health.sdu.dk. The imputed HLA alleles and corresponding parental age at death from the UK Biobank are available through application with a project proposal at https://www.ukbiobank.ac.uk/register-apply/. Additionally, the software used or any additional custom code for data processing and analysis is available at the corresponding sources mentioned in the Methods. Supplementary code is available in the GitHub repository (https://github.com/nmendozam/Mendoza-et-al.-2025-HLA-assoc) [71].

## Abbreviations

AD: Alzheimer’s disease
APOB-100: Apolipoprotein B-100
APOE: Apolipoprotein E
BCE: Before the common era
FOXO3: Forkhead box O3
GWAS: Genome-wide association study
HLA: Human leucocyte antigen
LLI: Long-lived individuals
IEDB: Immune epitope database
IKMB: Institute of Clinical Molecular Biology
OR: Odd ratio
*P*: *P*-Value
PheWAS: Phenome-wide association study
SE: Standard error
SNP: Single-nucleotide polymorphism
TCR: T-cell receptor
