# Supplementary material for: HLA-DRB1*15:01 is associated with a reduced likelihood of longevity in northern European men
Source: Genome Med. 2025 Oct 20;17:125. doi: 10.1186/s13073-025-01554-1 (PMC12539080; doi:10.1186/s13073-025-01554-1)
Supplement: Supplementary file 1 — Additional file 1: Contains the supplementary figures and methods. These include figures of distribution of parental age at death in the UK Biobank (Fig. S1); results of the quality control on individuals and markers (for the German dataset; Figs. S1, S2); other phenotypes associated with DRB1*15:01 identified by PheWAS (Fig. S3) and diagnostic plots for the immunogenicity logistic regression model (Fig. S4). The file also includes a description of the interaction tests (Methods S1) and two tables supplementing related to it (Tables S11, S12). [file 13073_2025_1554_MOESM1_ESM.docx]

**Supplementary Figures for**

**HLA-DRB1*15:01 is associated with a reduced likelihood of longevity in northern European men**

Nicolás Mendoza-Mejía¹, Daniel Kolbe¹, Onur Özer1, Janina Dose¹, Guillermo G. Torres¹, Andre Franke¹, Marianne Nygaard², Almut Nebel¹

¹ Institute of Clinical Molecular Biology, Kiel University, University Hospital Schleswig-Holstein, Kiel, Germany

² The Danish Twin Registry, University of Southern Denmark, Odense, Denmark

**Keywords:** human leukocyte antigen, longevity, genetic association study, ageing, immunogenetics


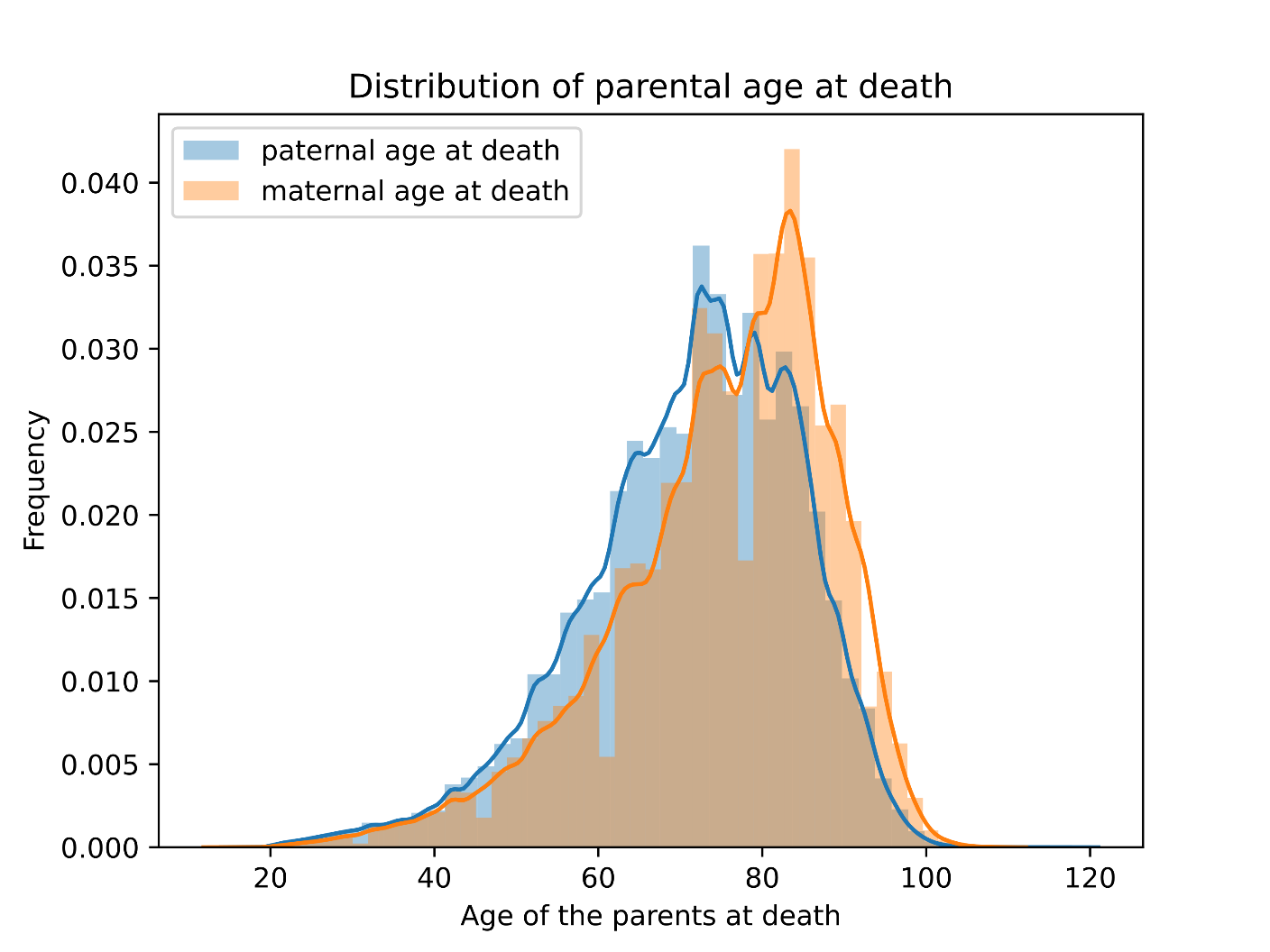


**Fig. S1: Distribution of parental age at death.**

The father’s and mother’s age-at-death distribution of samples from the UK Biobank. This distribution was used to determine the 99^th^ percentile threshold used for the long-lived parents.


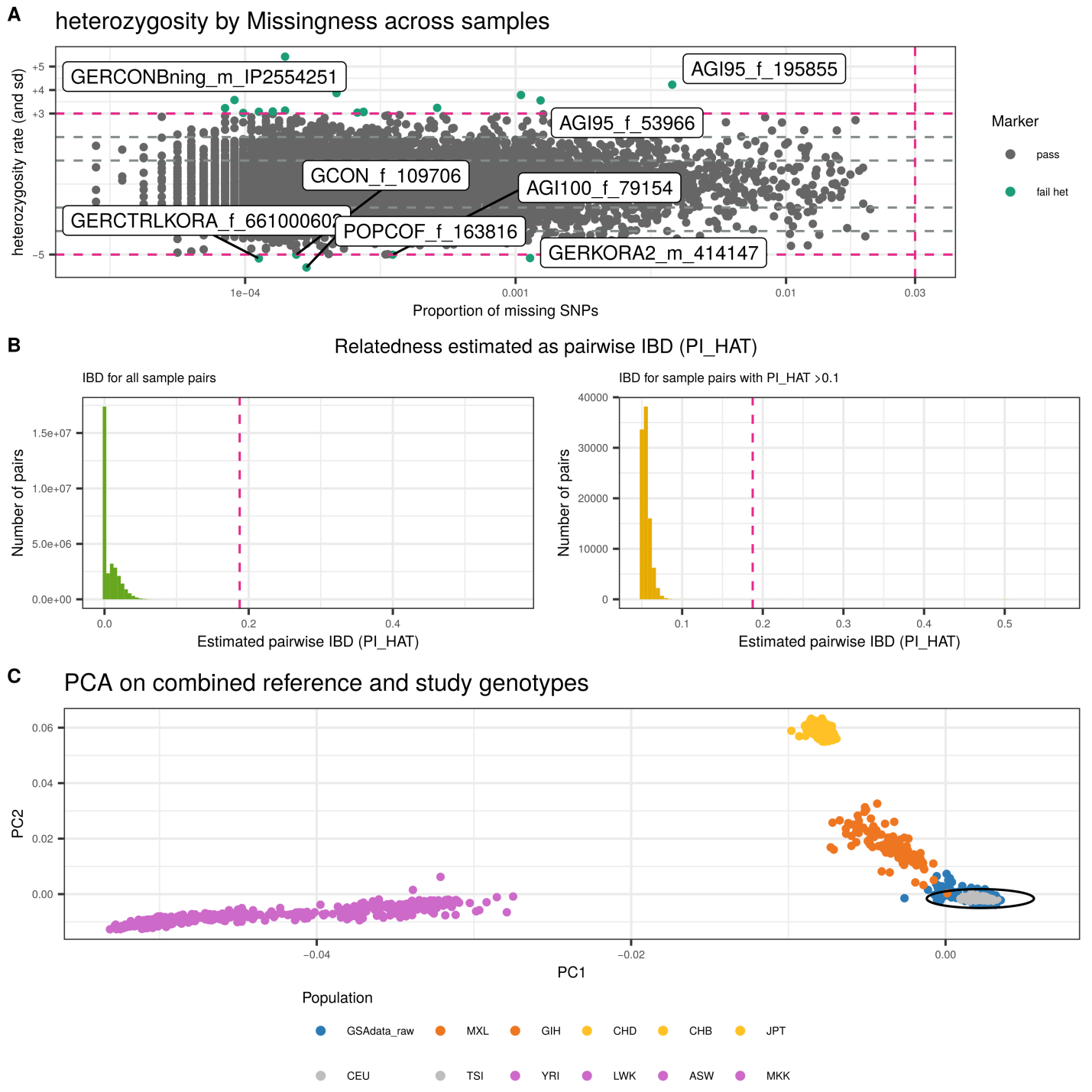


**Fig. S2: Individual-based quality control.**

Quality control steps applied to the German longevity cohort. All panels were generated with plinkQC. **A**: Heterozygosity vs. missingness rate plot, highlighting outlier samples (green dots) removed based on excessive missingness or heterozygosity (dashed red lines). **B**: Histograms of pairwise identity-by-descent (IBD) values, demonstrating the removal of related individuals based on a PI_HAT > 0.18 threshold (red line). **C**: Principal component analysis (PCA) plot, showing study samples (blue) clustering with the European CEU reference population (grey) and indicating the removal of individuals with divergent ancestry to ensure population homogeneity.


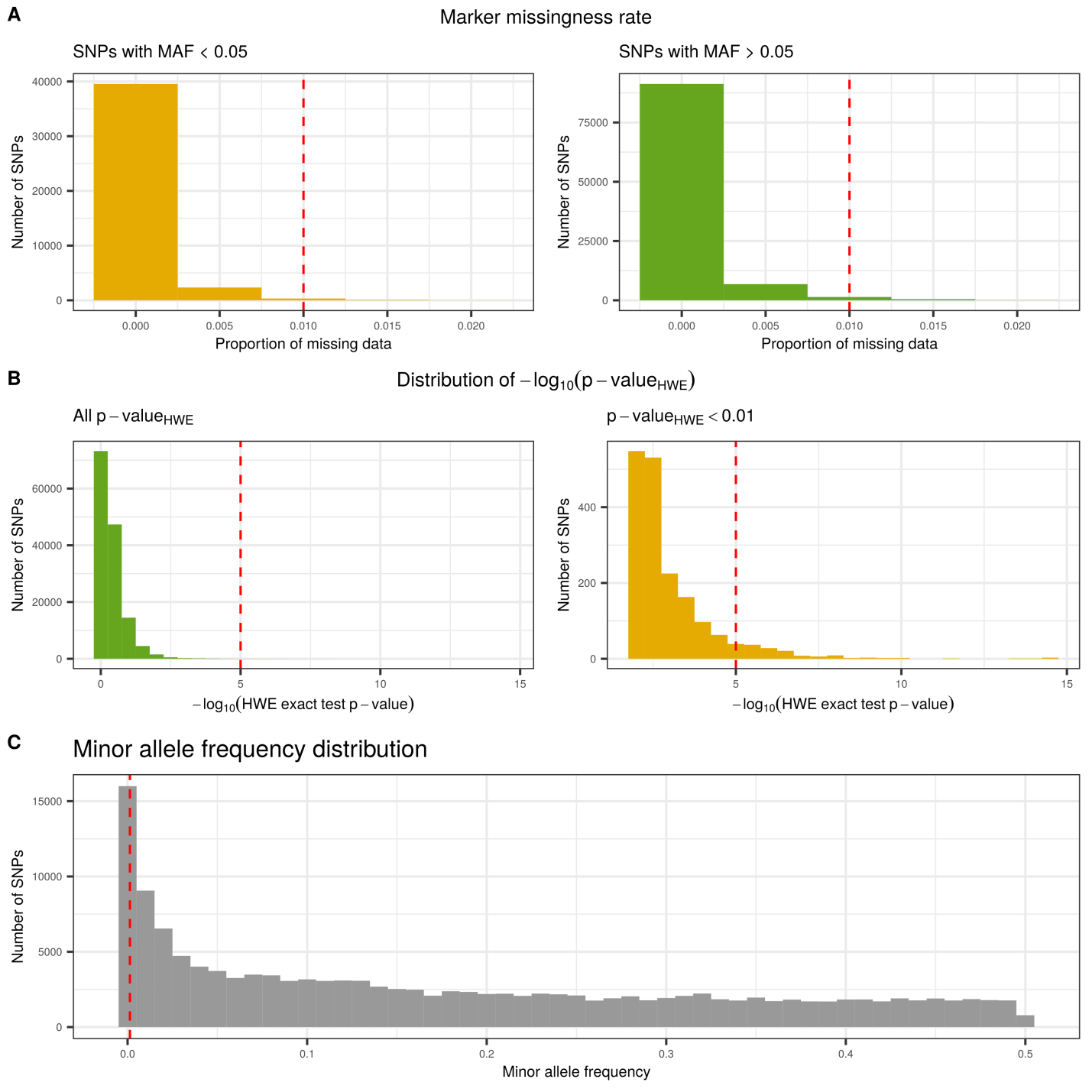


**Fig. S3: Quality control on markers.**

Quality control applied to genetic markers (SNPs). All panels were generated with plinkQC. **A**: Histograms of marker missingness rates, separated for SNPs with minor allele frequency (MAF) below and above 0.05. Markers exceeding a missingness threshold (red line) were removed. **B**: Histograms of the distribution of -log10(Hardy-Weinberg Equilibrium p-values) for all SNPs (left) and for SNPs with p-value_HWE < 0.01 (right). Markers deviating significantly from Hardy-Weinberg Equilibrium (p-value_HWE < 1x10^-05^, red line) were excluded. **C**: MAF distribution across all SNPs, with a red line indicating the minimum minor allele count threshold (<20) for marker retention.


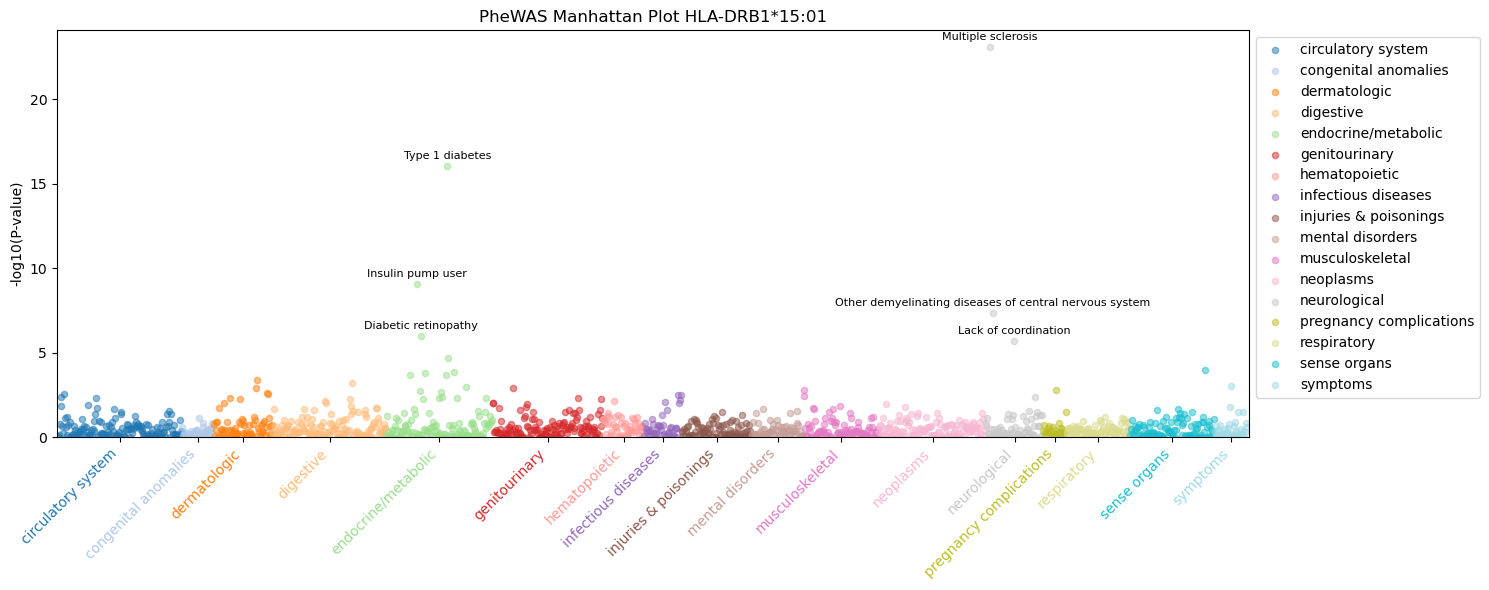


**Fig. S4: Phenome-wide association study (PheWAS) for *HLA-DRB1*15:01*.**

The Manhattan plot illustrates the strength of association (-log_10_ p-value) between *HLA-DRB1*15:01* and a wide range of phenotypes. Each point represents a phenotype, grouped by broad disease categories along the x-axis. Notably, the phenotype with the strongest association is multiple sclerosis, exhibiting a substantially more significant p-value compared to other associations, consistent with established literature. Data source: (https://phewascatalog.org/phewas/#hla, accessed March 25, 2025)


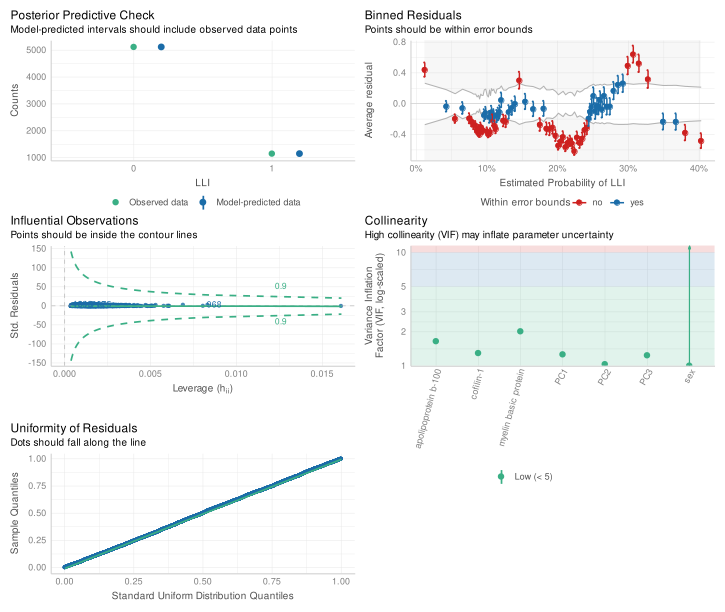


**Fig. S5 Visual inspection of the assumptions of the logistic regression model.**

Diagnostic plots for the logistic regression model were generated using check_model (from the R library “performance”). The model assessed the link between immunogenicity scores and longevity. The posterior predictive check shows a reasonable model fit and the binned residuals suggest that the linearity assumption was met. Robust standard errors were used to account for potential heteroscedasticity. The influential observations plot indicates no highly influential data points, and the collinearity plot shows variance inflation factors (VIF) within an acceptable range. Finally, the uniformity of residuals plot suggests that the residuals are reasonably uniformly distributed, collectively indicating that the assumptions of the logistic regression model are generally satisfied.

**Methods S1: Interaction tests**

This set of tests assesses independence, synergistic or antagonistic interactions, differential associations, combined action, and linkage disequilibrium between two genetic factors (A and B). These tests are commonly implemented in HLA association tools, where the genetic factors correspond to HLA alleles. The tests involve rearranging the 2×2 contingency tables (Table S12), which are subsequently analysed using Fisher’s exact test. The null hypothesis was rejected when P<0.05. An interaction between A and B was considered significant only when tests 1 and 3 were significant. Similarly, associations were considered independent when tests 1 and 2, or tests 3 and 4, were significant. Table S13 shows how to interpretate the results of the 8 interaction tests.

**Table S11:** **Number of alleles in cases and controls.** This table is describing the variables that will later be used in the contingency tables for the interaction tests. Factor A and B indicate the condition that should be met by the number of cases and controls. For example, X1 and Y1 are the number of cases and controls respectively that are both positive for factor A and B. In contrast, X2 and Y2 are the number of cases and controls that are positive for factor A, but negative for B.

|  |  | **Number of** | |
| --- | --- | --- | --- |
| **Factor A‍** | **Factor B** | **Cases** | **Controls** |
| +‍ | + | X1 | Y1 |
| +‍ | - | X2 | Y2 |
| -‍ | + | X3 | Y3 |
| -‍ | - | X4 | Y4 |

**Table S12: Contingency tables for interaction tests.** It describes how the contingency tables are constructed to perform the interaction analysis of two factors.

|  | **Entities of 2x2 contingency table** | | | |  |
| --- | --- | --- | --- | --- | --- |
| ‍‍‍‍‍‍‍‍‍‍**Comparison** | **A** | **B** | **C** | **D** | **Null hypothesis** |
| ‍‍‍‍‍‍‍‍‍‍++vs-+ | X1 | X3 | Y1 | Y3 | [1] A is not associated with the outcome. |
| ‍‍‍‍‍‍+-vs--‍ | X2 | X4 | Y2 | Y4 | [2] B is not associated with the outcome. |
| ‍‍‍‍‍‍++vs+- | X1 | X2 | Y1 | Y2 | [3] A is not associated with the outcome in B-positive individuals. |
| -+vs--‍‍ | X3 | X4 | Y3 | Y4 | [4] A is not associated with the outcome in B-negative individuals. |
|  |  |  |  |  |  |
| +-vs-+ | X2 | X3 | Y2 | Y3 | [5] There is no difference between the associations of A and B. |
| ++vs--‍ | X1 | X4 | Y1 | Y4 | [6] There is no combined association of A and B with the outcome. |
| Association between A and B cases | X1 | X2 | X3 | X4 | [7] A and B are not in linkage disequilibrium in cases. |
| Association between A and B controls | Y1 | Y2 | Y3 | Y4 | [8] A and B are not in linkage disequilibrium in controls. |

**Table S13. Questions asked with the interaction tests.** Since the interpretation of significant interactions is sometimes difficult, this table is included to list the possible questions that can be solved depending on which tests were significant and therefore rejected the null hypothesis.

| **Significant tests** | **Result** |
| --- | --- |
| Test [1] | A is associated with the phenotype in B-positives |
| Test [2] | A is associated with the phenotype in B-negatives |
| Test [1] + [2] | A is associated with the phenotype independently of B |
| Test [3] | B is associated with the phenotype in A-positives |
| Test [4] | B is associated with the phenotype in A-negatives |
| Test [3] + [4] | B is associated with the phenotype independently of A |
| Test [1] + [3] | A and B interact and are associated with the phenotype |
| Test [2] + [4] | A and B are associated with the phenotype only when the other factor is not present |
| Test [5] | The absence of A and B is associated with the phenotype |
| Test [6] | A and B have a combined association with the phenotype |
| Test [7] | There is linkage disequilibrium in cases between A and B |
| Test [8] | There is no linkage disequilibrium in controls between A and B |
